# Supplementary material for: Views of health professionals on risk-based breast cancer screening and its implementation in the Spanish National Health System: A qualitative discussion group study
Source: PLoS One. 2022 Feb 4;17(2):e0263788. doi: 10.1371/journal.pone.0263788 (PMC8815913; doi:10.1371/journal.pone.0263788)
Supplement: S1 Table — (DOCX) [file pone.0263788.s001.docx]

**S1 Table. Discussion groups guide**

| **Time** | **Outline** |
| --- | --- |
| 10 min | **Welcome, introduction of the participants, and thank you**  Introduction of the research team  Introduction of the participants: name, professional profile, workplace, work schedule  Thanks for participating and importance of their participation |
| 2 min | **General information on the topic to be addressed and purpose of the session**  *All of you have received an email inviting you to participate in a study of risk-based breast cancer screening and shared decision making. The goal of the study is to explore barriers and facilitators of coordination between levels of care in order to facilitate the implementation of such a screening program.*  *You all work in the healthcare system and directly or indirectly are, or would be, actors involved in the risk-based breast cancer screening program. For this reason, we believe it is essential to obtain your view and opinions.*  *The information provided here is confidential. This means that we are interested in exploring the different views on the subject, without paying attention on WHO has a specific point of view.* |
| 5 min | **Ethical aspects and confidentiality: requesting informed consent and permission to record**  *In order to analyze the content of the session, it is essential to record it, so we ask your permission to do so. The entire research team is committed to collecting the information, analyzing and preparing the results with confidentiality.* |
| 3 min | **Session dynamics and recommendations**  The moderators explain that the session is structured in two parts:  1) Two members of the research team explain to the participants what risk-based screening and shared decision-making consist of. The participants share their views in response to some open questions.  2) Participants are divided into two discussion groups to expose their opinions on barriers and facilitators in the different phases of the screening program.  The moderators propose some questions that participants can answer following an open and spontaneous format. They explain that all opinions are equally valid, that there are no right or wrong answers, and that it is very important that participants express their personal points of view. Moderators remark that it is important that everyone has the chance to talk and ask to avoid crossed conversations. |
| 20 min | **Contribution of the two members of the research team with slides support**  What is a risk-based breast cancer screening program?  Description of shared decision-making and shared decision-making within the risk-based screening program  Presentation of a proof of concept study, carried out by the research group, about the feasibility and acceptability of risk-based screening.  Moderators ask participants if they have questions or need any additional clarification. |
| 10 min | **General assessment of the risk-based breast cancer screening model**  Explore participants’ reactions to the research team’s contribution:  *What do you think?*  *Is it feasible to implement a risk-based breast cancer screening program from your professional position?* |
| 20 min | **Identification of barriers and facilitators when providing information to women on the risk-based screening program?**  From the proof of concept study presented, identify barriers and facilitators when providing information about the program to women.  *• The proposal made by the research team is that both Primary Care and Screening Program professionals inform about the program. From your experience what are the barriers, in general, that can make it difficult to inform women about the risk-based screening program?*  *• What are the coordination barriers between the different actors (primary care, screening program, breast unit, health managers) that may hinder the process of providing information about the risk-based breast cancer screening program?*  *• What coordination facilitators can help in providing information about the risk-based breast cancer screening program?*  *• What needs to be done to turn coordination barriers into facilitators?* |
| 20 min | **Identification of barriers and facilitators in women's risk assessment**  From the proof of concept study presented, identify barriers and facilitators to coordinate women’s risk assessment and information.  *What are the barriers, in general, that can make it difficult to assess a woman's risk?*  *What are the coordination barriers between the different actors (primary care, screening program, breast unit, health managers) that may hinder this process?*  *What are the coordination facilitators that can help in women’s risk assessment?*  *What needs to be done to turn coordination barriers into facilitators?* |
| 15 min | **Identification of barriers and facilitators in the screening recommendation based on the estimated risk, in a shared decision-making context**  From the proof of concept study presented, identify the barriers and facilitators of coordination in the professional’s recommendation based on the estimated risk, in a shared decision-making context.  *What are the barriers, in general, that can hinder the information and screening recommendation based on the estimated risk, in a shared decision-making context?*  *What are the coordination barriers between the different actors (primary care, screening program, breast unit, health managers) that may hinder this process?*  *What are the coordination facilitators that can help provide the information and screening recommendations based on the estimated risk, in a shared decision-making context?*  *What needs to be done to turn coordination barriers into facilitators?* |
| 5 min | **End of the meeting**  Moderators ask if participants would like to add anything  Conclusion  Acknowledgment for their participation |
